# GDF-15 and uEGF Independently Associate With CKD Progression in Children

**DOI:** 10.1016/j.ekir.2025.07.004

**Published:** 2025-07-16

**Authors:** Julia Bartels, Mansoureh Tabatabaeifar, Marietta Kirchner, Karolis Azukaitis, Anke Doyon, Aysun Bayazit, Ali Düzova, Nur Canpolat, Ipek Kaplan Bulut, Lukasz Obrycki, Bruno Ranchin, Rukshana Shroff, Francesca Lugani, Cengiz Candan, Jerome Harambat, Harika Alpay, Mieczyslaw Litwin, Augustina Jankauskiene, Günter Klaus, Dorota Drozdz, Giovanni Montini, Aleksandra Zurowska, Alberto Caldas Afonso, Elke Wühl, Anette Melk, Uwe Querfeld, Otto Mehls, Franz Schaefer, 4C Study and ESCAPE Trial Consortia, G. Cortina, K. Arbeiter, J. Dusek, J. Harambat, B. Ranchin, M. Fischbach, A.Zalosczyk, U. Querfeld, S.Habbig, M. Galiano, R. Büscher, C. Gimpel, M. Kemper, A. Melk, D. Thurn, F. Schaefer, A. Doyon, E. Wühl, M. Pohl, S. Wygoda, N. Jeck, B. Kranz, M. Wigger, G. Montini, F. Lugani, S. Testa, E. Vidal, C. Matteucci, S. Picca, A. Jankauskiene, K. Azukaitis, A. Zurowska, D. Drodz, M. Tkaczyk, T. Urasinski, M. Litwin, A.Niemirska, M. Szczepanska, A. Texeira, A. Peco-Antic, B.Bucher, G. Laube, A. Anarat, A.K. Bayazit, F. Yalcinkaya, E. Basin, N. Cakar, O. Soylemezoglu, A. Düzova, Y. Bilginer, H. Erdogan, O. Donmez, A. Balat, A. Kiyak, S. Caliskan, N. Canpolat, C. Candan, M. Civilibal, S. Emre, G. Ozcelik, S. Mir, B. Sözeri, O. Yavascan, Y. Tabel, P. Ertan, E. Yilmaz, R. Shroff

**Affiliations:** 1 Division of Pediatric Nephrology, Medical Faculty Heidelberg, Center for Pediatrics and Adolescent Medicine, Heidelberg University Hospital, Heidelberg University, Heidelberg, Germany; 2 Institute of Medical Biometry and Informatics, University of Heidelberg, Heidelberg, Germany; 3 Clinic of Pediatrics, Faculty of Medicine, Institute of Clinical Medicine, Vilnius University, Vilnius, Lithuania; 4 Department of Pediatric Nephrology, Cukurova University, Adana, Türkiye; 5 Division of Pediatric Nephrology, Hacettepe University Faculty of Medicine, Ankara, Türkiye; 6 Istanbul University Cerrahpasa Medical Faculty, Istanbul, Türkiye; 7 Ege University Faculty of Medicine, Izmir, Türkiye; 8 Department of Nephrology, Kidney Transplantation and Arterial Hypertension, The Children’s Memorial Health Institute, Warsaw, Poland; 9 Pediatric Nephrology Unit, Hôpital Femme Mère Enfant, Hospices Civils de Lyon, Université de Lyon, Lyon, France; 10 Department of Pediatric Nephrology, UCL Great Ormond Street Hospital and Institute of Child Health, London, UK; 11 Department of Pediatric Nephrology, Istituto Gaslini, Genoa, Italy; 12 Göztepe Educational and Research Hospital, Istanbul, Türkiye; 13 Department of Pediatrics, Bordeaux University Hospital, Bordeaux, France; 14 Department of Pediatric Nephrology, Marmara University Faculty of Medicine, Istanbul, Türkiye; 15 Department of General Pediatrics, Philipps-University, Marburg, Germany; 16 Department of Pediatric Nephrology and Hypertension, Pediatric Institute, Jagiellonian University Medical College, Cracow, Poland; 17 Fondazione IRCCS Ca' Granda Ospedale Maggiore Policlinico, Pediatric Nephrology, Dialysis and Transplant Unit, Milan, Italy; 18 Department of Clinical Sciences and Community Health, University of Milan, Milan, Italy; 19 Department Pediatrics, Nephrology & Hypertension, Medical University Gdansk, Gdansk, Poland; 20 Centro Materno Infantil do Norte, Centro Hospitalar do Porto, Abel Salazar Biomedical Sciences Institute, University of Porto, Porto, Portugal; 21 Department of Kidney, Liver and Metabolic Diseases, Hannover Medical School, Hannover, Germany; 22 Charité Childrenʼs Hospital, Berlin, Germany

**Keywords:** chronic kidney disease, CKD progression, epidermal growth factor, growth differentiation factor 15, pediatric CKD

## Abstract

**Introduction:**

Currently, there is limited ability to predict the progression of chronic kidney disease (CKD) in children. Previously we reported that low levels of urinary epidermal growth factor (uEGF) predict CKD progression in children. In the present study, we investigated a novel serum biomarker, growth differentiation factor 15 (GDF-15), in 2 European pediatric CKD cohorts. We additionally explored the combined effect of GDF-15 and/or uEGF on CKD progression in children.

**Methods:**

The association between serum GDF-15 levels and CKD progression was analyzed in 671 patients of the Cardiovascular Comorbidity in Children with CKD (4C) study, aged 6 to 17 years with an estimated glomerular filtration rate (eGFR) of 10 to 60 ml/min per 1.73 m^2^ at baseline, and median follow-up of 8 years. The composite end point was start of kidney replacement therapy, 50% eGFR loss, or eGFR < 10 ml/min per 1.73 m^2^. Results were validated in 329 participants from the ESCAPE trial.

**Results:**

Higher GDF-15 levels were associated with an increased risk of CKD progression (hazard ratio: 1.40; 95% confidence interval [CI]: 1.10–1.77), independent of age, sex, baseline eGFR, proteinuria, and systolic blood pressure. Whereas adding either GDF-15 or uEGF individually to a model containing these variables improved model fit, combining both markers improved the model further. External validation in the ESCAPE cohort confirmed these results.

**Conclusion:**

Serum GDF-15 and urine EGF levels may provide complementary information on the risk of CKD progression in children and might be included in future prognostic biomarker panels aimed at personalized, risk-stratified management of pediatric CKD.

## Graphical abstract

**Figure:**
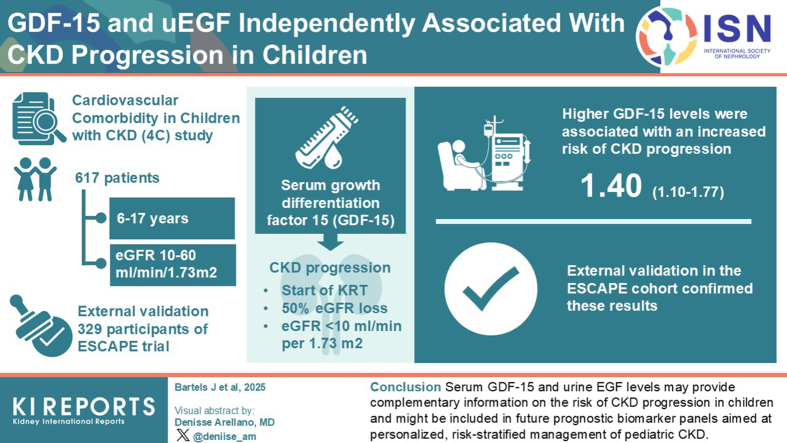

CKD in children and adolescents leads to important clinical sequelae, including impaired neurocognitive performance, reduced quality of life, and an increased risk of cardiovascular disease.^1^, ^2^, ^3^, ^4^, ^5^ The rate of disease progression varies greatly between and even within individuals^6^^,^^7^ and typically accelerates as the children approach kidney failure.^8^

Reliable biomarkers predicting the risk of CKD progression would help develop personalized, risk-adapted nephroprotective strategies. However, despite ample research, few biomarkers exceeding the prognostic value of serum creatinine and proteinuria have been identified to date.^9^^,^^10^

uEGF has been identified as an independent biomarker of CKD progression both in adults^11^ and in 2 pediatric cohorts.^12^ uEGF levels appear to reflect the integrity of the tubulointerstitial compartment, with low concentrations reflecting tubular atrophy and interstitial fibrosis.

The limited number of validated biomarkers highlights the need for additional markers that reflect diverse pathways of CKD progression.

GDF-15, a protein from the TGF-ß superfamily, is a promising candidate biomarker of CKD progression. It is a ligand for the brain-specific α-like receptor of the Glial-Derived Neurotrophic Factor family and was proposed to be a ligand of the ErbB2 receptor as well, activating the ARK and the ERK-1/2 signalling pathways.^13^^,^^14^ Expression and plasma concentrations are regulated by p53,^15^ EGR-1,^16^ CHOP, and ATF4^17^, ^18^, ^19^; therefore, GDF-15 is upregulated in response to tissue injury, inflammation and ischemia.^20^ It is expressed almost ubiquitously; in the kidney, expression increases in response to harmful processes such as metabolic acidosis or potassium depletion.^21^, ^22^, ^23^, ^24^ Previous studies found GDF-15 to play a role in cardioprotection.^21^^,^^23^^,^^24^ A recent preclinical study indicated that GDF-15 might also be nephroprotective, as GDF-15 knockout mice showed increased interstitial and tubular damage in models of type 1 and type 2 diabetes.^25^ An association of GDF-15 with the incidence and progression of CKD has recently been observed in adults. In adults without known kidney disease followed in the Framingham Heart study, higher serum GDF-15 concentrations were associated with a greater risk of developing CKD.^26^ In 2 adult CKD cohorts, intrarenal GDF-15 mRNA expression correlated with circulating serum GDF-15 levels, which in turn were associated with CKD progression.^27^ However, after adjustment for cardiovascular and age-related risk factors the association was only weakly significant.

Data on GDF-15 in children with kidney disease are scarce. Elevated serum GDF-15 levels have been reported in pediatric patients on dialysis,^28^ but its role as a biomarker of CKD progression in children remains unclear.

Here, we investigated the association of serum GDF-15 levels alone and in combination with uEGF with the risk of CKD progression in 2 large European pediatric CKD cohorts. The Cardiovascular Comorbidity in Children with CKD (4C) Study cohort was used to explore associations and develop a prediction model, which was subsequently validated in the ESCAPE trial cohort.

## Methods

### Study Design and Setting

The 4C Study is a long-term observational cohort study that followed 704 children with CKD in 12 European countries for up to 8 years. Participants were aged 6 to 17 years with a baseline eGFR of 10 to 60 ml/min per 1.73 m^2^ at the time of enrolment and underwent semiannual clinical assessments from 2010 to 2018. The detailed study design and setting were described previously.^29^ The ESCAPE trial was a randomized controlled clinical trial addressing the nephroprotective effect of intensified blood pressure control.^30^ Three hundred eighty-five European children aged 3 to 17 years were followed for 5 years, with 2-monthly eGFR assessments. Baseline eGFR ranged from 15 to 80 ml/min per 1.73 m^2^. All ESCAPE patients received a fixed dose of the angiotensin-converting enzyme inhibitor ramipril throughout the observation period.

Patients from both cohorts were included if a frozen serum sample collected at baseline or within 6 months of enrolment were available to assess serum GDF-15 levels, along with available data on CKD progression. In addition, for a subset of patients, previously measured uEGF concentrations from one morning urine sample collected at baseline or within the first 6 months of enrolment were available and included in the analysis. Both study protocols were approved by the central ethics committee of the Medical Faculty of Heidelberg University and by each of the local institutional review boards.

### Laboratory Assessments

Samples were aliquoted and stored at −80 °C directly after collection. Urine and serum samples were analyzed centrally in a standardized manner as published in the study protocols.^5^^,^^29^^,^^30^ eGFR was determined based on serum creatinine and cystatin C,^31^ and proteinuria according to the urine protein-to-creatinine ratio (mg/mg).

For GDF-15 measurements in serum, the Human GDF-15 Quantikine enzyme-linked immunosorbent assay (R&D Systems, Minneapolis, MN) was used as previously described.^27^ All samples were determined in duplicate wells. Only samples collected within 6 months of enrolment were used. uEGF was measured in urine spot samples as previously described.^12^

### Clinical Assessments

In accordance with the study protocol, anthropometric and clinical data were collected at each study visit.^5^ Weight, height, and blood pressure were normalized to standard deviation scores (SDS).^32^

### Statistical Analysis

Patient baseline characteristics are described separately for the 4C and ESCAPE cohort using mean with SD, median with interquartile range, or frequencies. Correlations were quantified using the Spearman correlation coefficient. Primary kidney diseases were categorized into congenital anomalies of the kidney and the urinary tract (CAKUT), tubulointerstitial disease, glomerulopathy, CKD after acute kidney injury, and others. The primary end point was defined as a composite event of 50% loss of baseline eGFR, attainment of an eGFR < 10 ml/min per 1.73 m^2^, or the start of kidney replacement therapy, whichever occurred first. If a 50% loss of eGFR occurred between 2 clinical visits, the time of the event was determined using interpolation. Survival analysis was performed by defining GDF-15 quartiles and comparing the respective progression-free survival. The log-rank test was applied and Kaplan-Meier survival curves are plotted. Furthermore, Cox proportional hazard models were applied to quantify the additional value of GDF-15 to predict progression of CKD. First, a model with basic prognostic variables was fitted (model 0) containing age, (biological) sex, systolic blood pressure SDS, log-transformed eGFR, log-transformed urine protein-to-creatinine ratio, and diagnosis (glomerulopathies vs. others); and second, log-transformed GDF-15 was added (model 1).

For a subgroup of patients with both GDF-15 and uEGF measured, models 0 and 1 (here model 1a) were applied as described above, and a third Cox proportional hazard model (model 2) was constructed by adding log-transformed uEGF to model 1. Ain addition, a model 1b was constructed by adding uEGF but not GDF-15 to model 0. All models were compared based on the Akaike information criterion (AIC, lower values indicating a better fit). C-statistics were used to evaluate the performance of the prediction models with GDF-15 using the method proposed by Uno *et al.*^33^ A truncated C (Cτ) was calculated with the R package survC1 for τ = 1, 2, and 3 years.^34^ The difference of the C-statistics of the models was calculated with 95% CIs to compare the predictive power of the models. A 95% CI not including 0 indicates significant improvement.

Internal validation was performed by calculating leave-one-out cross-validation and bootstrap corrected estimates. External validation was performed in the ESCAPE dataset. The developed model from the 4C dataset was applied to the validation dataset as follows. After fitting the Cox proportional hazards model to the 4C data, the model coefficient estimates were used to calculate the linear predictor of the existing model for the patients of the ESCAPE cohort. For this purpose, the “predict” function in R with the mean centered linear predictor method (type="lp") was used. Here, too, truncated C-statistics were calculated. A calibration plot was created to compare the predicted survival probability in the ESCAPE cohort with the observed survival frequencies.

A *P*-value < 0.05 was considered significant. Statistical analysis was performed using SAS Software Version 9.4 (SAS Inc., Cary, NC) or R version 4.0.1.

## Results

### Description of Cohorts

Serum samples were available from 671 children in the 4C study and from 329 participants in the ESCAPE trial. The baseline characteristics of both cohorts are shown in Table 1.

**Table 1:** Baseline characteristics of the 4C and ESCAPE cohorts

Baseline characteristics	4C cohort	ESCAPE cohort
Total, *N*	671	329
Male sex (%)	434 (65.1)	129 (58.4)
Age, mean (SD), yrs	12.2 (3.4)	11.6 (4.0)
Primary renal diagnosis (%)		
CAKUT	462 (69.3)	237 (72.0)
Glomerulopathies	57 (8.5)	22 (6.7)
Post-AKI CKD	33 (4.9)	30 (9.1)
Tubulointerstitial disorders	89 (13.3)	33 (10.0)
Other	26 (3.9)	7 (2.1)
Systolic blood pressure		
mm Hg	112 (14.8)	118 (14.4)
SDS	0.74 (1.35)	0.77 (1.21)
Diastolic blood pressure		
mm Hg	68.6 (12.2)	72.4 (12.3)
SDS	0.63 (1.07)	1.23 (1.24)
Body mass index		
kg/m^2^	18.5 (3.9)	18.1 (3.6)
SDS	0.12 (1.24)	−0.19 (1.11)
Height		
cm	141 (20.2)	140 (22.4)
SDS	−1.35 (1.36)	−1.43 (1.51)
eGFR, ml/min per 1.73 m^2^	27.6 (13.4)	44.5 (18.0)
uEGF/Cr^a^, ng/mg	3.58 (4.69)	4.38 (4.99)
uPCR^a^, mg/mg	1.29 (2.45)	0.90 (1.71)
GDF-15^a^, pg/ml	1004 (708)	805 (487)
Total cholesterol, mg/dl	181 (51.6)	NA
LDL cholesterol, mg/dl	99.8 (42.4)	NA
HDL cholesterol, mg/dl	48.0 (14.9)	NA
Plasma bicarbonate, mmol/l	21.2 (3.8)	22.6 (3.7)
Serum albumin, g/l	39 (6.3)	42.8 (6.0)
CRP^a^, mg/l	0.58 (1.99)	NA

AKI, acute kidney injury; CAKUT, congenital anomalies of the kidney and the urinary tract; CKD, chronic kidney disease; CRP, C-reactive protein; eGFR, estimated glomerular filtration rate; GDF-15, growth differentiation factor 15; HDL, high-density lipoprotein; LDL, low-density lipoprotein; NA, not applicable; SDS, SD scores; uEGF/Cr, urinary epidermal growth factor / urinary creatinine; uPCR, urine protein-to-creatinine ratio.

Data are *N* (%) and mean (SD).

a The variables are given as median (interquartile range).

The leading primary renal diagnoses in the 4C cohort were CAKUT (69.3%), tubulointerstitial diseases (13.3%), and glomerulopathies (8.5%). Of the 4C patients, 63.6% received renin-angiotensin system inhibitors during the observation period. In the ESCAPE trial cohort, primary renal diagnoses were distributed similarly with 72% CAKUT and 7% glomerulopathies. The ESCAPE patients were on average younger and presented with higher eGFR and lower proteinuria (0.90 [1.71] mg/mg) than the 4C patients (Table 1).

In the 4C cohort, the composite end point of CKD progression was reached by 389 patients (58%) after a median time of 2.3 (0.8–4.4) years, corresponding to an incidence rate of 20.8 events per 100 patient-years. In the ESCAPE cohort, 121 (36.8%) patients reached the primary end point during the 5-year follow-up.

### Serum GDF-15 Levels

The distribution of serum concentration of GDF-15 in the 2 cohorts is presented in Table 1. In both cohorts, GDF-15 was inversely correlated with eGFR (4C: *r* = −0.58, *P* < 0.001; ESCAPE: *r* = −0.66, *P* < 0.001). A multivariable linear regression model was constructed on the 4C cohort to identify covariates of GDF-15. Log-transformed GDF-15 was associated with lower eGFR (ß = −0.02, *P* < 0.001), lower serum albumin (ß = −0.02, *P* < 0.001), higher C-reactive protein (ß = 0.05, *P* < 0.001) and the diagnoses (reference: CAKUT) glomerulopathies (ß = 0.18, *P* 0.004), tubulointerstitial disorders (ß = 0.28, *P* < 0.001), and others (ß = 0.31, *P* < 0.001) but not with age, sex, body mass index SDS, and uPCR.

### Serum GDF-15 and CKD Progression

**Figure 1:**
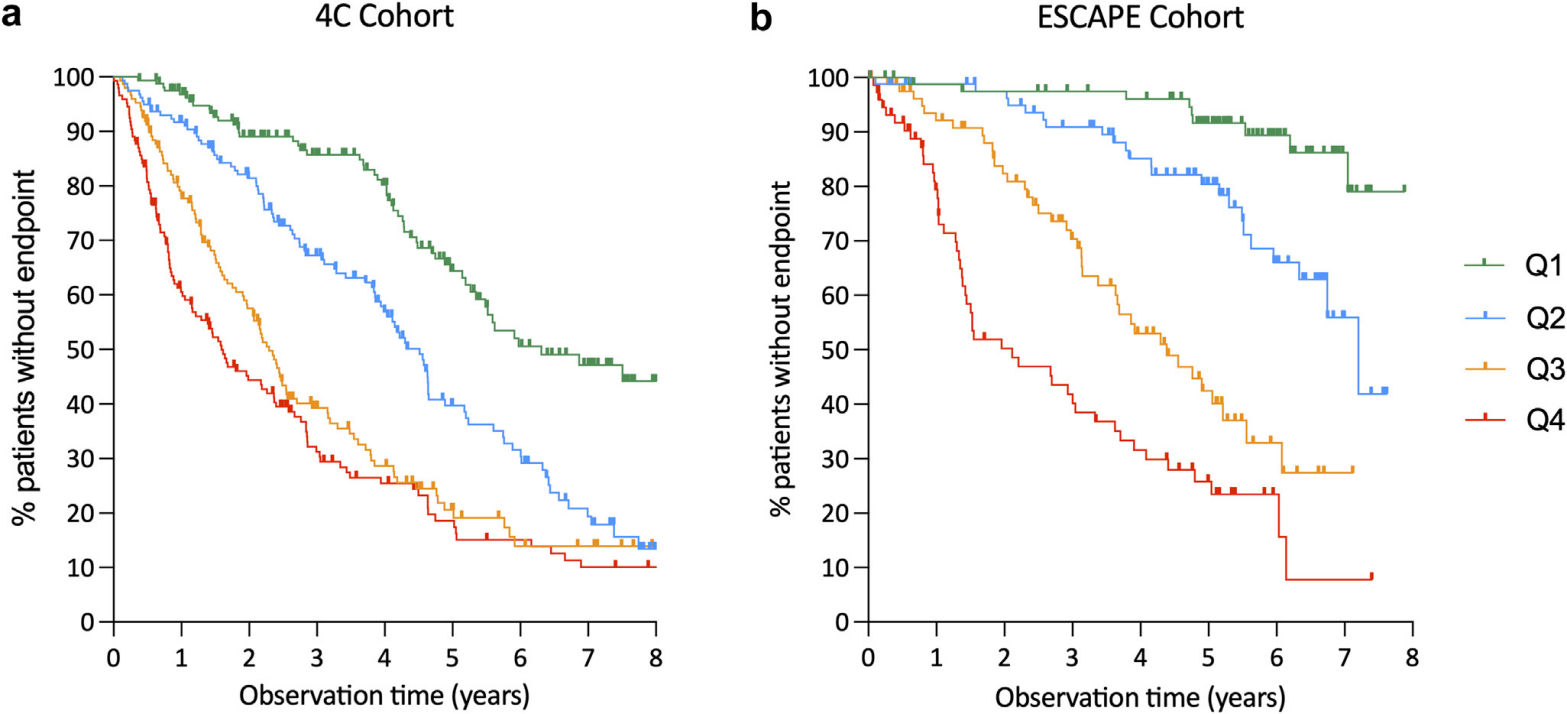
CKD progression-free kidney survival by serum GDF-15 quartiles in 4C cohort (a) and ESCAPE cohort (b). CKD, chronic kidney disease; GDF-15, growth differentiation factor 15.

In both cohorts, progression-free survival was quantitatively associated with serum GDF-15 levels. Patients with GDF-15 concentrations in the highest quartile showed the highest risk of CKD progression (log-rank test with *P* < 0.001 for between-group differences, Figure 1).

Cox proportional hazard models with established progression markers without and with serum GDF-15 were constructed (model 0 and model 1, Table 2 and Supplementary Table S1). In model 0 for the 4C cohort, log-transformed eGFR and log-transformed urine protein-to-creatinine ratio, age, systolic blood pressure SDS, and glomerulopathy (vs. others) were significantly associated with CKD progression. In model 1, log-transformed GDF-15 showed a significant independent association with CKD progression (hazard ratio: 1.40, 95% CI: 1.10–1.77, *P* = 0.006) and improved the predictive value of the model based on the AIC (Table 2). C-statistics were calculated to compare the predictive models. The addition of GDF-15 to model 0 improved C-statistics for predicting 1-, 2-, and 3-year risk of CKD progression at borderline significance (95% CI: −0.001 to 0.02, 95% CI: −0.001 to 0.01 and 95% CI: −0.001 to 0.01 at 1, 2, and 3 years respectively) (Table 3). The results of the C-statistics were validated internally by leave-one-out cross-validation and bootstrapping, which also showed improved values for risk progression at all time points.

**Table 2:** Cox proportional hazard models of CKD progression

Variable	Model 0 (AIC 4063)				Model 1 (AIC 4058)			
	Hazard ratio	95% CI		*P*-value	Hazard ratio	95% CI		*P*-value
Sex (female)	1.07	0.87	1.32	0.51	1.05	0.85	1.30	0.65
Age (yrs)	1.06	1.02	1.09	< 0.001	1.05	1.02	1.09	< 0.001
Glomerulopathies	1.64	1.16	2.32	0.005	1.53	1.08	2.18	0.02
log uPCR	1.51	1.39	1.66	< 0.001	1.50	1.37	1.64	< 0.001
Systolic BP SDS	1.11	1.04	1.20	0.003	1.10	1.03	1.19	0.007
log eGFR	0.14	0.11	0.19	< 0.001	0.17	0.12	0.23	< 0.001
log GDF-15	-	-	-	-	1.40	1.10	1.77	0.006

AIC, Akaike information criterion; CI, confidence interval; CKD, chronic kidney disease; eGFR, estimated glomerular filtration rate; GDF-15, growth differentiation factor 15; SDS, SD score; uPCR, urinary protein-to-creatinine ratio.

*n* = 667; reference for glomerulopathies: other diagnoses.

**Table 3:** C-statistics and internal validation of CKD prediction model incorporating GDF-15 at different time points in the 4C cohort

Time point	C-statistic (95% CI)			Leave-1-out cross-validation		Bootstrapping	
	Model 0	Model 1	ΔC-statistic	Model 0	Model 1	Model 0	Model 1
1-yr	0.851 (0.815–0.887)	0.858 (0.825–0.892)	0.007 (−0.001 to 0.015)	0.849	0.855	0.849	0.855
2-yr	0.814 (0.785–0.844)	0.819 (0.790–0.847)	0.004 (−0.001 to 0.010)	0.812	0.815	0.812	0.815
3-yr	0.799 (0.769–0.828)	0.803 (0.774–0.831)	0.004 (−0.001 to 0.010)	0.796	0.799	0.796	0.800

CI, confidence interval; CKD, chronic kidney disease; eGFR, estimated glomerular filtration rate; GDF-15, growth differentiation factor 15; SDS, SD score; uPCR, urinary protein-to-creatinine ratio.

Model 0 includes sex, age, log-transformed uPCR, systolic blood pressure SDS, and log-transformed eGFR. Model 1 includes all variables of model 0 and log-transformed GDF-15.

**Figure 2:**
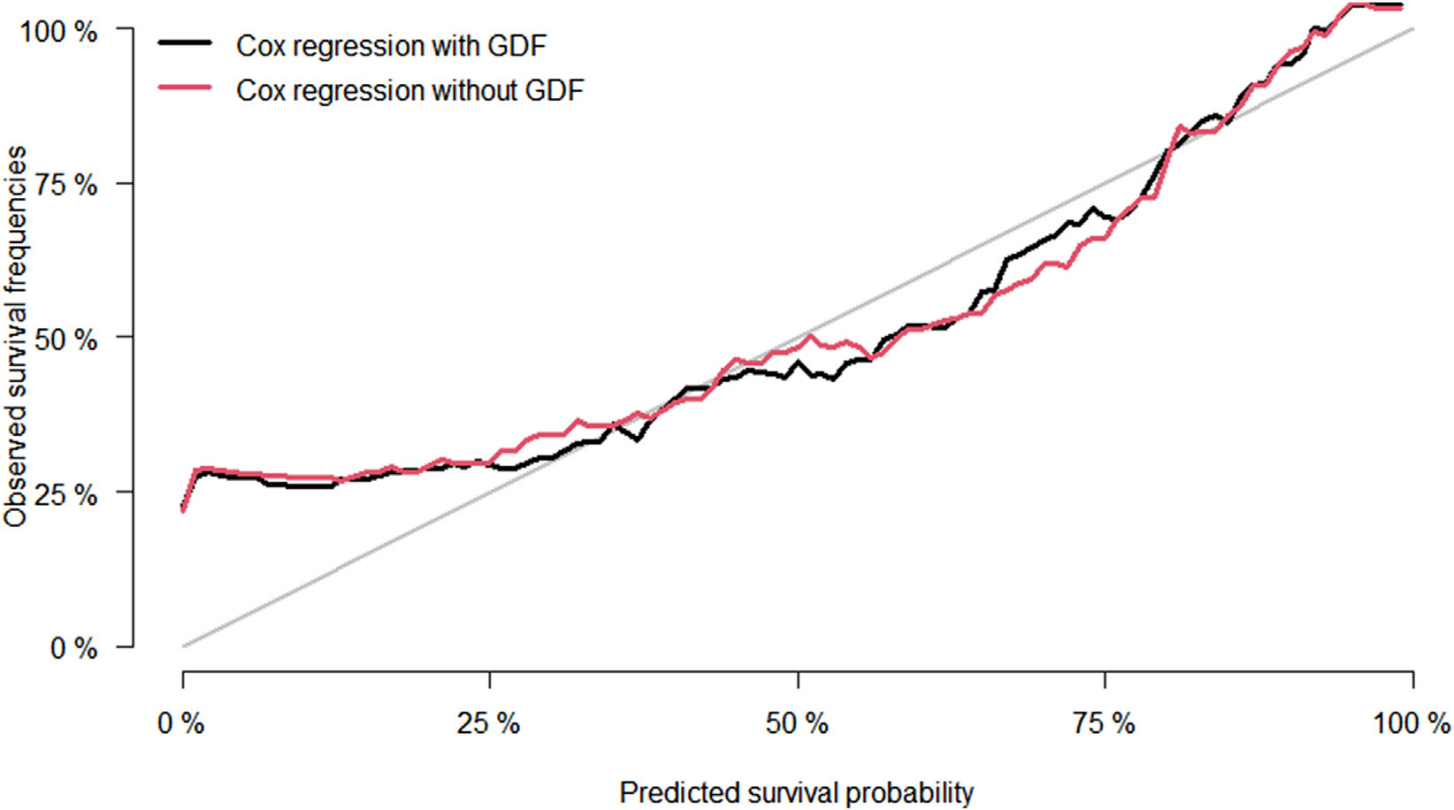
Calibration plot of predicted and observed survival probability in the ESCAPE validation cohort. GDF, growth differentiation factor.

In the ESCAPE cohort, serum GDF-15 concentrations were also associated with CKD progression independently of eGFR, proteinuria, and other risk factors (hazard ratio: 4.49, 95% CI: 2.35–8.58, *P* < 0.001) (Supplementary Table S1). C-statistics were nominally increased by addition of log-transformed GDF-15 levels to the model, approaching statistical significance with increasing observation time (95% CI: −0.04 to 0.08, 95% CI: −0.003 to 0.06, and 95% CI: −0.001 to 0.05 at 1, 2, and 3 years respectively) (Table 4). A calibration plot of the predicted and the observed survival probability is shown in Figure 2. The predicted survival probability was well in line with the observed survival frequencies across most of the probability distribution range except for the lower end, where survival was underpredicted in the ESCAPE validation cohort.

**Table 4:** External validation of CKD progression prediction model incorporating GDF-15: model fit (C-Statistic) in ESCAPE cohort

Time point	Model 0	Model 1	ΔC-statistic
1-yr	0.828 (0.713–0.943)	0.847 (0.741–0.954)	0.019 (−0.042 to 0.080)
2-yr	0.864 (0.808–0.919)	0.889 (0.840–0.939)	0.026 (−0.003 to 0.055)
3-yr	0.845 (0.791–0.899)	0.869 (0.824–0.915)	0.024 (−0.001 to 0.050)

### GDF-15 and uEGF Combined

In subgroups of 536 4C and 183 ESCAPE patients with serum GDF-15 measurements, urinary EGF concentrations were available. Among those children, 299 4C (55.7%) and 70 ESCAPE (38.3%) patients reached the composite end point. Four Cox proportional hazard models were constructed; models 0 and 1(a) as described earlier, a model lb with the baseline values and uEGF (without GDF-15), and a model containing both log-transformed GDF-15 and log-transformed uEGF (model 2).

Both GDF-15 and uEGF were significantly associated with CKD progression in the 4C cohort independently of the conventional risk factors established in model 0 (Table 5, model 1a and 1b). The fit of the model according to the AIC improved when either GDF-15 or uEGF were added to the model (3069 to 3062 and 3053, respectively). Adding both markers together further improved the model fit (AIC 3051).

**Table 5:** Cox proportional hazard models with stepwise inclusion of GDF-15 and uEGF in the 4C cohort

Variable	Model 0 (AIC 3069)				Model 1a (AIC 3062)			
	Hazard ratio	95% CI		*P*	Hazard ratio	95% CI		*P*
Sex (female)	1.09	0.86	1.39	0.48	1.06	0.83	1.35	0.64
Age (yrs)	1.07	1.03	1.11	< 0.001	1.07	1.03	1.10	< 0.001
Glomerulopathies	1.73	1.16	2.59	0.01	1.56	1.03	2.34	0.03
log uPCR	1.55	1.40	1.71	< 0.001	1.53	1.38	1.69	< 0.001
Systolic BP SDS	1.13	1.03	1.24	0.01	1.12	1.02	1.22	0.02
log eGFR	0.22	0.16	0.31	< 0.001	0.27	0.18	0.38	< 0.001
log GDF-15	-	-	-	-	1.47	1.14	1.90	0.003
log uEGF/Cr	-	-	-	-	-	-	-	-

Variable	**Model 1b (AIC 3053)**				**Model 2 (AIC 3051)**			
	Hazard ratio	95% CI		*P*	Hazard ratio	95% CI		*P*
Sex (female)	1.10	0.87	1.41	0.42	1.09	0.85	1.38	0.51
Age (yrs)	1.05	1.01	1.09	0.007	1.05	1.02	1.09	0.006
Glomerulopathies	1.61	1.08	2.42	0.02	1.49	0.99	2.26	0.06
log uPCR	1.72	1.53	1.93	<0.001	1.68	1.50	1.89	< 0.001
Systolic BP SDS	1.14	1.04	1.24	0.007	1.13	1.03	1.23	0.01
log eGFR	0.30	0.21	0.44	< 0.001	0.33	0.23	0.49	< 0.001
log GDF-15	-	-	-	-	1.31	1.01	1.71	0.05
log uEGF/Cr	0.81	0.74	0.89	< 0.001	0.83	0.75	0.91	< 0.001

AIC, Akaike information criterion; BP, blood pressure; CI, confidence interval; eGFR, estimated glomerular filtration rate; GDF-15, growth differentiation factor 15; SDS, SD score; uEGF/Cr, urinary epidermal growth factor / urinary creatinine; uPCR, urinary protein-to-creatinine ratio.

*n* = 536; reference for Glomerulopathies: other diagnoses.

In the ESCAPE cohort, the results of the Cox models were consistent with those obtained in the 4C cohort. When adjusting for age, sex, systolic blood pressure SDS, proteinuria, diagnosis, and eGFR, higher serum GDF-15 was associated with a 4-fold increased hazard of reaching the end point (model 1a; *P* < 0.001). In model 1b, higher uEGF levels were associated with a 39% risk reduction (*P* = 0.007). The combination of GDF-15 and uEGF (model 2) showed further improvement based on the AIC (587, 576, 582, and 574 for models 0, 1a, 1b, and 2, respectively) as compared with each biomarker alone (Supplementary Table S2).

## Discussion

The aim of this investigation was to explore whether serum GDF-15 levels are associated with the risk of CKD progression in children and whether a combination of GDF-15 and urinary EGF provides better prediction of CKD progression than each factor alone. We demonstrated an association between GDF-15 concentrations and the progression of pediatric CKD, in keeping with findings in adults.^26^^,^^27^ The association was independent of established progression factors. Internal and external validation confirmed these findings. However, the observed association was not close enough to allow risk discrimination in a prediction model at the given sample size, with C-statistics reaching borderline statistical significance.

We aimed to identify a reliable predictive marker for pediatric CKD progression. Biomarkers should ideally be related to the pathophysiology of the disease, that is, be biologically plausible.^35^ Several findings indicate the involvement of GDF-15 in the progression of CKD. GDF-15 is weakly expressed along the whole nephron and upregulated in response to damaging processes such as metabolic acidosis.^22^ Intrarenal expression of GDF-15 was strongly correlated with serum GDF-15 levels in adults with CKD.^27^

This is the first time the association of serum GDF-15 with CKD progression was investigated in large cohorts of children with CKD. Pediatric CKD differs profoundly from adult disease, as it is caused predominantly by congenital and hereditary disorders and patients largely lack comorbid conditions related to aging, diabetes, and smoking. Since serum GDF-15 reflects various stressors in the body and is therefore associated with aging and disease,^36^ studying it in pediatric cohorts may reduce confounding and allow a clearer assessment of its association with CKD progression.

Data on GDF-15 in children with kidney disease have been limited and inconclusive. In a small cohort of children on chronic dialysis, serum GDF-15 levels were consistently elevated compared to healthy controls, with higher levels in children receiving hemodialysis than in those on peritoneal dialysis.^28^ In contrast, urinary GDF-15 showed little or no correlation with kidney function in pediatric allograft recipients.^37^ Moreover, although serum GDF-15 was studied as a biomarker for cardiovascular disease in children, it was found unsuitable for that purpose because of its strong association with kidney function.^37^

To assess whether GDF-15 is associated with progression of CKD independently of established progression factors, baseline models for the Cox proportional hazard analyses were constructed by including risk factors according to previous studies.^38^ A clear association of GDF-15 with the risk of CKD progression was observed, which remained significant after adjusting for these established risk factors. Most importantly, the addition of GDF-15 to the model improved the AIC, indicating a better fit of the model. For 1-, 2-, and 3-year risk of progression, the C-statistics nominally increased at all times (indicating improvement) and in both the internal and external validation studies; however, only borderline statistical significance was reached.

These findings suggest that though GDF-15 is independently associated with CKD progression in children, its added prognostic value as a single biomarker appears to be modest. It has been argued that panels of biomarkers might be more informative on CKD progression than single markers.^35^ uEGF has been identified as a promising biomarker of CKD progression in children.^12^ In a subgroup of patients for whom both GDF-15 and uEGF measurements were available, the addition of each marker to the baseline model improved the fit of the model based on the AIC. Adding both GDF-15 and uEGF to the baseline model resulted in an even further improvement of the model fit. Therefore, the combination of GDF-15 and uEGF may provide complementary information on the risk of CKD progression in children.

The cross-validation of findings in 2 large prospective pediatric CKD cohorts is a major strength of our study, which covered a wide range of primary kidney diseases and ethnic backgrounds. Nonetheless, both the study cohort and the validation cohort lacked representation of children with early CKD (stages 1 and 2), infant age, as well as African American and Asian backgrounds. Our investigation was further limited by the fact that only one-time measurements of both biomarkers were performed, precluding assessment of the association of changes in serum levels with the risk of CKD progression. In addition, GDF-15 and uEGF could not always be measured in samples from the same visit, although all samples were obtained within a 6-month time window. The measurement of GDF-15 in urine instead of serum samples could provide additional information because GDF-15 levels may be related with uremic toxin levels and this relation is less with urinary GDF-15.^39^ Furthermore, the treatment effect of angiotensin-converting enzyme inhibitors on GDF-15 and uEGF levels could be investigated in future studies.

In conclusion, GDF-15 is associated with the progression of CKD in children independent of established variables and across all primary renal disorders including CAKUT. While isolated serum GDF-15 levels yield a marginal improvement of progression prediction models, the combination of GDF-15 and uEGF appears to provide complementary information on disease progression, and thus both markers are potential candidates for a biomarker panel.

Further exploration of GDF-15, uEGF, and other potential biomarkers of CKD progression in large multiethnic prospective pediatric cohorts including younger patients and children with earlier stages of CKD will be needed to corroborate and extend our findings with the aim of establishing reliable biomarker panels that will help personalize nephroprotective management for children with CKD, such as frequency of patient monitoring and timing of implementation of pharmacological therapies, according to individual risk.

## Appendix

### List of the Members of the 4C Study and ESCAPE Trial Consortia

Austria: G. Cortina, Children’s Hospital, Innsbruck; K. Arbeiter, University Children’s Hospital, Vienna. Czech Republic: J. Dusek, University Hospital Motol, Prague. France: J. Harambat, Hôpital des Enfants, Bordeaux; B. Ranchin, Hôpital Femme Mère Enfant et Université de Lyon; M. Fischbach, A.Zalosczyk, Hôpital de Hautepierre, Strasbourg. Germany: U. Querfeld, Charité Children’s Hospital, Berlin; S.Habbig, University Children’s Hospital, Cologne; M. Galiano, University Children’s Hospital, Erlangen; R. Büscher, University Children’s Hospital, Essen; C. Gimpel, Center for Pediatrics and Adolescent Medicine, Freiburg; M. Kemper, UKE University Children’s Hospital, Hamburg; A. Melk, D. Thurn, Hannover Medical School, Hannover; F. Schaefer, A. Doyon, E. Wühl, Center for Pediatrics and Adolescent Medicine, Heidelberg; M. Pohl, Center for Pediatrics and Adolescent Medicine, Jena; S. Wygoda, City Hospital St. Georg, Leipzig; N. Jeck, KfH Kidney Center for Children, Marburg; B. Kranz, University Children’s Hospital, Münster; M. Wigger, Children’s Hospital, Rostock. Italy: G. Montini, S. Orsola-Malpighi Hospital, Bologna; F. Lugani, Istituto Giannina Gaslini, Genova; S. Testa, Fondazione Ospedale Maggiore Policlinico, Milano; E. Vidal, Pediatric Nephrology, Dialysis & Transplant Unit, Padova; C. Matteucci, S. Picca, Ospedale Bambino Gesù, Rome. Lithuania: A. Jankauskiene, K. Azukaitis, University Children’s Hospital, Vilnius. Poland: A. Zurowska, Pediatric and Adolescent Nephrology, Gdansk; D. Drodz, University Children’s Hospital, Krakow; M. Tkaczyk, Polish Mothers Memorial Hospital Research Institute, Lodz; T. Urasinski, Clinic of Pediatrics, Szczecin; M. Litwin, A.Niemirska, Children’s Memorial Health Institute, Warsaw; M. Szczepanska, Zabrze. Portugal: A. Texeira, Hospital Sao Joao, Porto; Serbia: A. Peco-Antic, University Children’s Hospital, Belgrade. Switzerland: B.Bucher, Inselspital, Bern; G. Laube, University Children’s Hospital, Zurich. Türkiye: A. Anarat, A.K. Bayazit, Cukurova University, Adana; F. Yalcinkaya, Ankara University Faculty of Medicine, Ankara; E. Basin, Baskent University Faculty of Medicine, Ankara; N. Cakar, Diskapi Children’s Hospital, Ankara; O. Soylemezoglu, Gazi University Hospital, Ankara; A. Düzova, Y. Bilginer, Hacettepe University Faculty of Medicine, Ankara; H. Erdogan, Dortcelik Children’s Hospital, Bursa; O. Donmez, Uludag University, Bursa; A. Balat, University of Gaziantep; A. Kiyak, Bakirkoy Children’s Hospital, Istanbul; S. Caliskan, N. Canpolat, Istanbul University Cerrahpasa Faculty of Medicine, Istanbul; C. Candan, Goztepe Educational and Research Hospital, Istanbul; M. Civilibal, Haseki Educational and Research Hospital, Istanbul; S. Emre, Istanbul Medical Faculty, Istanbul, H. Alpay, Marmara University Medical Faculty, Istanbul; G. Ozcelik, Sisli Educational and Research Hospital, Istanbul; S. Mir, B. Sözeri, Ege University Medical Faculty; Izmir; O. Yavascan, Tepecik Training and Research Hospital, Izmir; Y. Tabel, Inonu University, Malatya; P. Ertan, Celal Bayar University, Manisa; E. Yilmaz, Children’s Hospital, Sanliurfa. United Kingdom: R. Shroff, Great Ormond Street Hospital, London.

## Disclosure

All the authors declared no competing interests.

## Supplementary Material

Supplementary File (PDF) **Table S1.** Cox proportional hazard models of CKD progression in the ESCAPE cohort. **Table S2.** Cox proportional hazard models with stepwise inclusion of GDF-15 and uEGF in the ESCAPE cohort. TRIPOD Checklist.

## Footnotes

Supplementary File (PDF)
**Table S1.** Cox proportional hazard models of CKD progression in the ESCAPE cohort. **Table S2.** Cox proportional hazard models with stepwise inclusion of GDF-15 and uEGF in the ESCAPE cohort. TRIPOD Checklist
