# Supplementary material for: GDF-15 and uEGF Independently Associate With CKD Progression in Children
Source: Kidney Int Rep. 2025 Jul 16;10(10):3369–78. doi: 10.1016/j.ekir.2025.07.004 (PMC12545905; doi:10.1016/j.ekir.2025.07.004)
Supplement: Supplementary File (PDF) — Table S1. Cox proportional hazard models of CKD progression in the ESCAPE cohort. Table S2. Cox proportional hazard models with stepwise inclusion of GDF-15 and uEGF in the ESCAPE cohort. TRIPOD Checklist. [file mmc1.pdf]

SUPPLEMENTARY MATERIAL

Table S1: Cox proportional hazard models of CKD progression in the ESCAPE cohort.

	Model 0 (AIC 819)				Model 1 (AIC 801)			
	**Hazard Ratio**	**95% CI**		***P* value**	**Hazard Ratio**	**95% CI**		***P* value**
**Sex (female)**	1.17	0.76	1.80	0.48	1.13	0.74	1.75	0.57
**Age (years)**	1.06	1.01	1.12	0.02	1.02	0.97	1.08	0.45
**Glomerulopathies**	1.64	0.89	3.02	0.11	1.35	0.73	1.75	0.57
**log uPCR**	1.36	1.10	1.69	0.004	1.36	1.10	1.67	0.004
**Systolic BP SDS**	1.11	0.94	1.32	0.22	1.06	0.89	1.26	0.54
**log eGFR**	0.09	0.06	0.16	<0.001	0.19	0.10	0.34	<0.001
**log GDF-15**	-	-	-	-	4.49	2.35	8.58	<0.001

n=262; reference for Glomerulopathies: other diagnoses; Abbreviations: CKD, chronic kidney disease; AIC, Akaike information criterion; CI, confidence interval; uPCR, urinary protein-to-creatinine ratio; SDS, SD score; eGFR, estimated glomerular filtration rate; GDF-15, growth differentiation factor-15.

Table S2: Cox proportional hazard models with stepwise inclusion of GDF-15 and uEGF in the ESCAPE cohort.

	Model 0 (AIC 587)				Model 1a (AIC 576)			
	Hazard Ratio	95% CI		*P*	Hazard Ratio	95% CI		*P*
**Sex (female)**	1.18	0.71	1.93	0.53	1.21	0.73	1.98	0.46
**Age (years)**	1.09	1.02	1.16	0.01	1.05	0.98	1.23	0.16
**Glomerulopathies**	1.80	0.89	3.64	0.10	1.63	0.80	3.30	0.18
**log uPCR**	1.26	0.99	1.60	0.06	1.30	1.01	1.66	0.04
**Systolic BP SDS**	1.14	0.95	1.37	0.16	1.09	0.91	1.32	0.36
**log eGFR**	0.11	0.06	0.20	<0.001	0.21	0.10	0.43	<0.001
**log GDF-15**	-	-	-	-	4.00	1.93	8.26	<0.001
**log uEGF/Cr**	-	-	-	-	-	-	-	-

	Model 1b (AIC 582)				Model 2 (AIC 574)			
	Hazard Ratio	95% CI		*P*	Hazard Ratio	95% CI		*P*
**Sex (female)**	1.18	0.72	1.93	0.52	1.22	0.74	1.99	0.44
**Age (years)**	1.03	0.96	1.11	0.38	1.01	0.94	1.09	0.77
**Glomerulopathies**	1.53	0.75	3.13	0.24	1.45	0.71	2.98	0.31
**log uPCR**	1.30	1.03	1.63	0.03	1.32	1.04	1.67	0.02
**Systolic BP SDS**	1.15	0.96	1.37	0.14	1.09	0.91	1.31	0.35
**log eGFR**	0.17	0.08	0.34	<0.001	0.29	0.13	0.64	0.002
**log GDF-15**	-	-	-	-	3.63	1.69	7.80	<0.001
**log uEGF/Cr**	0.61	0.43	0.88	0.007	0.68	0.46	0.99	0.04

n=183; reference for Glomerulopathies: other diagnoses; Abbreviations: AIC, Akaike information criterion; CI, confidence interval; uPCR, urinary protein-to-creatinine ratio; BP, blood pressure; SDS, SD score; eGFR, estimated glomerular filtration rate; GDF-15, growth differentiation factor-15; uEGF/Cr, urinary epidermal growth factor/urinary creatinine**.**

4C Study Collaborators

The following principal investigators contributed to the 4C Study:

Austria: G. Cortina, Children's Hospital, Innsbruck; K. Arbeiter, University Children's Hospital, Vienna. **Czech Republic:** J. Dusek, University Hospital Motol, Prague. **France:** J. Harambat, Hôpital des Enfants, Bordeaux; B. Ranchin, Hôpital Femme Mère Enfant et Université de Lyon; M. Fischbach, A.Zalosczyk, Hôpital de Hautepierre, Strasbourg. **Germany:** U. Querfeld, Charité Children's Hospital, Berlin; S.Habbig, University Children's Hospital, Cologne; M. Galiano, University Children's Hospital, Erlangen; R. Büscher, University Children's Hospital, Essen; C. Gimpel, Center for Pediatrics and Adolescent Medicine, Freiburg; M. Kemper, UKE University Children's Hospital, Hamburg; A. Melk, D. Thurn, Hannover Medical School, Hannover; F. Schaefer, A. Doyon, E. Wühl, Center for Pediatrics and Adolescent Medicine, Heidelberg; M. Pohl, Center for Pediatrics and Adolescent Medicine, Jena; S. Wygoda, City Hospital St. Georg, Leipzig; N. Jeck, KfH Kidney Center for Children, Marburg; B. Kranz, University Children's Hospital, Münster; M. Wigger, Children's Hospital, Rostock. **Italy:** G. Montini, S. Orsola-Malpighi Hospital, Bologna; F. Lugani, Istituto Giannina Gaslini, Genova; S. Testa, Fondazione Ospedale Maggiore Policlinico, Milano; E. Vidal, Pediatric Nephrology, Dialysis & Transplant Unit, Padova; C. Matteucci, S. Picca, Ospedale Bambino Gesù, Rome. **Lithuania:** A. Jankauskiene, K. Azukaitis, University Children's Hospital, Vilnius. **Poland:** A. Zurowska, Pediatric and Adolescent Nephrology, Gdansk; D. Drodz, University Children's Hospital, Krakow; M. Tkaczyk, Polish Mothers Memorial Hospital Research Institute, Lodz; T. Urasinski, Clinic of Pediatrics, Szczecin; M. Litwin, A.Niemirska, Children's Memorial Health Institute, Warsaw; M. Szczepanska, Zabrze. **Portugal:** A. Texeira, Hospital Sao Joao, Porto; **Serbia:** A. Peco-Antic, University Children's Hospital, Belgrade. **Switzerland:** B.Bucher, Inselspital, Bern; G. Laube, University Children's Hospital, Zurich. **Türkiye:** A. Anarat, A.K. Bayazit, Cukurova University, Adana; F. Yalcinkaya, Ankara University Faculty of Medicine, Ankara; E. Basin, Baskent University Faculty of Medicine, Ankara; N. Cakar, Diskapi Children's Hospital, Ankara; O. Soylemezoglu, Gazi University Hospital, Ankara; A. Düzova, Y. Bilginer, Hacettepe University Faculty of Medicine, Ankara; H. Erdogan, Dortcelik Children's Hospital, Bursa; O. Donmez, Uludag University, Bursa; A. Balat, University of Gaziantep; A. Kiyak, Bakirkoy Children's Hospital, Istanbul; S. Caliskan, N. Canpolat, Istanbul University Cerrahpasa Faculty of Medicine, Istanbul; C. Candan, Goztepe Educational and Research Hospital, Istanbul; M. Civilibal, Haseki Educational and Research Hospital, Istanbul; S. Emre, Istanbul Medical Faculty, Istanbul, H. Alpay, Marmara University Medical Faculty, Istanbul; G. Ozcelik, Sisli Educational and Research Hospital, Istanbul; S. Mir, B. Sözeri, Ege University Medical Faculty; Izmir; O. Yavascan, Tepecik Training and Research Hospital, Izmir; Y. Tabel, Inonu University, Malatya; P. Ertan, Celal Bayar University, Manisa; E. Yilmaz, Children's Hospital, Sanliurfa. **United Kingdom:** R. Shroff, Great Ormond Street Hospital, London.

Reporting checklist for prediction model development/validation.

Based on the TRIPOD guidelines.

		Reporting Item	Page Number
**Title**			
	#1	Identify the study as developing and / or validating a multivariable prediction model, the target population, and the outcome to be predicted.	1
**Abstract**			
	#2	Provide a summary of objectives, study design, setting, participants, sample size, predictors, outcome, statistical analysis, results, and conclusions.	3
**Introduction**			
	#3a	Explain the medical context (including whether diagnostic or prognostic) and rationale for developing or validating the multivariable prediction model, including references to existing models.	4
	#3b	Specify the objectives, including whether the study describes the development or validation of the model or both.	4
**Methods**			
Source of data	#4a	Describe the study design or source of data (e.g., randomized trial, cohort, or registry data), separately for the development and validation data sets, if applicable.	4
Source of data	#4b	Specify the key study dates, including start of accrual; end of accrual; and, if applicable, end of follow-up.	4-5
Participants	#5a	Specify key elements of the study setting (e.g., primary care, secondary care, general population) including number and location of centres.	4-5
Participants	#5b	Describe eligibility criteria for participants.	4-5
Participants	#5c	Give details of treatments received, if relevant	4-5
Outcome	#6a	Clearly define the outcome that is predicted by the prediction model, including how and when assessed.	5
Outcome	#6b	Report any actions to blind assessment of the outcome to be predicted.	n/a
Predictors	#7a	Clearly define all predictors used in developing or validating the multivariable prediction model, including how and when they were measured	5
Predictors	#7b	Report any actions to blind assessment of predictors for the outcome and other predictors.	n/a
Sample size	#8	Explain how the study size was arrived at.	5

Missing data	#9	Describe how missing data were handled (e.g., complete-case analysis, single imputation, multiple imputation) with details of any imputation method.	5
Statistical analysis methods	#10a	If you are developing a prediction model describe how predictors were handled in the analyses.	5
Statistical analysis methods	#10b	If you are developing a prediction model, specify type of model, all model-building procedures (including any predictor selection), and method for internal validation.	5
Statistical analysis methods	#10c	If you are validating a prediction model, describe how the predictions were calculated.	5-6
Statistical analysis methods	#10d	Specify all measures used to assess model performance and, if relevant, to compare multiple models.	5-6
Statistical analysis methods	#10e	If you are validating a prediction model, describe any model updating (e.g., recalibration) arising from the validation, if done	5-6
Risk groups	#11	Provide details on how risk groups were created, if done.	5
Development vs. validation	#12	For validation, identify any differences from the development data in setting, eligibility criteria, outcome, and predictors.	5-6
**Results**			
Participants	#13a	Describe the flow of participants through the study, including the number of participants with and without the outcome and, if applicable, a summary of the follow-up time. A diagram may be helpful.	6
Participants	#13b	Describe the characteristics of the participants (basic demographics, clinical features, available predictors), including the number of participants with missing data for predictors and outcome.	6
Participants	#13c	For validation, show a comparison with the development data of the distribution of important variables (demographics, predictors and outcome).	7
Model development	#14a	If developing a model, specify the number of participants and outcome events in each analysis.	6
Model development	#14b	If developing a model, report the unadjusted association, if calculated between each candidate predictor and outcome.	6
Model specification	#15a	If developing a model, present the full prediction model to allow predictions for individuals (i.e., all regression coefficients, and model intercept or baseline survival at a given time point).	6
Model specification	#15b	If developing a prediction model, explain how to the use it.	n/a
Model performance	#16	Report performance measures (with CIs) for the prediction model.	6-7

Model-updating	#17	If validating a model, report the results from any model updating, if done (i.e., model specification, model performance).	6-7
**Discussion**			
Limitations	#18	Discuss any limitations of the study (such as nonrepresentative sample, few events per predictor, missing data).	7-8
Interpretation	#19a	For validation, discuss the results with reference to performance in the development data, and any other validation data	7-8
Interpretation	#19b	Give an overall interpretation of the results, considering objectives, limitations, results from similar studies, and other relevant evidence.	7-8
Implications	#20	Discuss the potential clinical use of the model and implications for future research	7-8
**Other information**			
Supplementary information	#21	Provide information about the availability of supplementary resources, such as study protocol, Web calculator, and data sets.	8-9
Funding	#22	Give the source of funding and the role of the funders for the present study.	12

The TRIPOD checklist is distributed under the terms of the Creative Commons Attribution License CC-BY. This checklist was completed on 12. April 2025 using https://www.goodreports.org/, a tool made by the EQUATOR Network in collaboration with Penelope.ai
